# 2845. Two Times versus Four Times Daily Cephalexin Dosing for the Treatment of Uncomplicated Urinary Tract Infection in Females

**DOI:** 10.1093/ofid/ofad500.2455

**Published:** 2023-11-27

**Authors:** Aidan Yetsko, Kristen Eid, Heather Draper, Andrew Jameson, Lisa E Dumkow

**Affiliations:** Trinity Health Grand Rapids, Grand Rapids, Michigan; Trinity Health Grand Rapids, Grand Rapids, Michigan; Trinity Health Grand Rapids, Grand Rapids, Michigan; Trinity Health Grand Rapids, Grand Rapids, Michigan; Trinity Health Grand Rapids, Grand Rapids, Michigan

## Abstract

**Background:**

The current IDSA treatment guidelines recommend beta-lactam antibiotics as an alternative, rather than first-line agents, for the treatment of uncomplicated UTI (uUTI). Changing susceptibilities over the past decade, however, have resulted in the need to rely on oral cephalosporins for uUTI. Cephalexin is a commonly prescribed oral first-generation cephalosporin with excellent bioavailability and urinary penetration; however, little data exist to support optimal dosing for uUTI, with pharmacokinetic data supporting twice daily dosing, but common drug databases recommending four times daily dosing.

**Methods:**

This retrospective, multicenter, cohort study included adult female patients treated at Trinity Health Michigan ambulatory sites who received cephalexin for symptomatic uUTI with cefazolin-susceptible urine cultures between February 1, 2020, and August 31, 2022. The primary objective was to compare uUTI treatment failure between patients treated with cephalexin 500 mg twice daily (BID Tx) versus 500 mg four times daily (QID Tx) in the outpatient setting. Treatment failure was defined as the combined endpoint of continued symptoms or necessitation of new therapy while on the initially prescribed 5-7 day course of treatment or recurrent UTI within 30 days of initial therapy. Secondary outcomes included reported adverse events within 7 days of treatment and occurrence of C. difficile within 30 days of treatment. Pregnancy, diagnosis of pyelonephritis or complicated UTI, presence of urinary catheter, history of recurrent UTI, receipt of IV antibiotics, or urine culture not susceptible to cefazolin resulted in exclusion.

**Results:**

A total of 261 patients were included (BID Tx, n= 173; QID Tx, n= 88). There was no difference in treatment failure observed between groups (BID Tx 12.7% vs QID Tx 17%, p=0.343), including failure while on therapy (BID Tx 2.3% vs QID Tx 5.7%, p=0.438) or recurrence within 30 days (BID Tx 10.4% vs QID Tx 11.3%, p=0.438). No differences in reported adverse events (BID Tx 4.6% vs QID Tx 5.7%, p=0.103) or C. difficile within 30 days of treatment (BID Tx 0% vs QID Tx 0%, p=1.0) were observed between groups.

**Conclusion:**

Reduced frequency, 500 mg twice daily, cephalexin is as effective as more frequent, four times daily, dosing for uUTI and may improve patient adherence.

**Disclosures:**

**All Authors**: No reported disclosures

